# Gene therapy for spinal muscular atrophy: timing is key

**DOI:** 10.1016/j.lanepe.2024.101112

**Published:** 2024-10-21

**Authors:** Laurent Servais

**Affiliations:** a MDUK Oxford Neuromuscular Centre, Department of Paediatrics, University of Oxford and NIHR Oxford Biomedical Research Center, Oxford OX3 9DU, United Kingdom; b Neuromuscular Center of Liège, Division of Paediatrics, CHU and University of Liège, 1 Bvd du XII de Ligne, Liège 4000, Belgium

Disease-modifying treatments have transformed the management and prognosis of spinal muscular atrophy (SMA) over the last eight years. Since the very first phase III trial conducted in SMA type 1,^1^ it became clear that the timing of treatment is key to ensuring the best efficacy. This is not surprising, as SMA is caused by the death of alpha motoneurons that do not replicate. The goal of treatments is to halt the irreversible mechanism of degeneration in motoneurons that are still alive. Additionally, these findings confirmed a large body of evidence from animal models.^2^

In this issue of *The Lancet Regional Health—Europe*, Weiβ and colleagues^3^ presents a very large cohort of 343 infants living with SMA in Germany, Austria and Switzerland and treated with gene therapy, significantly surpassing the largest national and international cohorts.^4^ In addition to its size, this cohort is well-followed and documented, making it an invaluable source of information for understanding the effects of treatments in SMA. It also gathers data on populations not represented in industry-funded trials, such as patients up to 90 months old at the time of treatment. The study brings additional strong evidence that the age at which the treatment delivered constitutes a crucial factor to optimise motor, respiratory and nutritional outcomes and to increase safety of innovative and costly medication such as gene therapy.

The fast-growing body of evidence in humans supported the rapid development of newborn screening (NBS) programs.^5^^,^^6^ Today, about 7% of babies worldwide are screened for SMA, and more than 1000 babies have been identified through NBS.^5^ Recently, the SMArtCARE and Swiss-Reg-NMD registries provided clear comparisons of motor, respiratory and nutritional outcomes for children born in regions where SMA is screened at birth versus those born in regions without screening.^7^ The overwhelming difference should strongly encourage decision-makers in countries like France and the UK, where SMA NBS lags behind countries like Ukraine and Poland, which have been screening for several years.^5^^,^^8^

Interestingly, the study also shows the incidence of hepatic adverse events caused by gene therapy increases with age at treatment, which further decreases the benefit-risk ratio of gene therapy in older patients. It would be interesting to study severe adverse reactions in the future, such as acute liver failure or long-term elevation of liver enzymes leading to the need for immunosuppression. Mild elevation of liver enzymes is not typically considered an adverse event when weighing the benefits and risks of gene therapy.

Several unanswered questions remain, and it is likely that only larger data collection and longer-term follow-up will provide answers. The first and foremost question is the comparison between the three different disease-modifying treatments, a comparison that will be challenging because the population of patients for whom they are prescribed as a first-line option differs significantly. Second, there is the question of the interest in switching or adding medication. Weiβ and colleagues did not find any “pre-treatment effect,” but pre-treatment before gene therapy can be prescribed under very different conditions, ranging from bridging therapy in neonates identified by NBS and presenting a high level of anti-AAV antibodies, to much older patients who have already been treated with risdiplam and/or nusinersen.^9^ The key question for physicians involved in daily patient management is whether there is a potential benefit in switching symptomatic patients on nusinersen or risdiplam to gene therapy and how this could affect their trajectories. The SMArtCARE and Swiss-Reg-NMD registries likely include enough data to answer this question.

The two final unanswered questions are, “How long?” How long do we collectively need to initiate NBS for SMA across Europe, given the tremendous amount of evidence that early treatment is better tolerated, much more effective, and massively cost-saving^6^^,^^7^^,^^10^? And finally, what have we learned from the spinal muscular atrophy journey that could be helpful for future conditions like Duchenne Muscular Dystrophy or Angelman syndrome, two conditions in which early-phase clinical trials have raised huge expectations in the community? Our readiness for NBS- our capacity to safely deliver innovative medication and to prospectively collect high-quality real-world data - certainly constitute part of the answer. The paper by Weiβ and colleagues^3^ provides additional and important evidence in this direction.

## Declaration of interests

LS has given consultancy/lectures for Biogen, Novartis, Roche, BioHaven, Scholar Rock, Zentech and Sysnav. He is part of the Data Safety Monitoring Board of NMB biopharma.
